# Efficacy and Safety of Adalimumab Biosimilars: Current Critical Clinical Data in Rheumatoid Arthritis

**DOI:** 10.3389/fimmu.2021.638444

**Published:** 2021-04-06

**Authors:** XiaoQin Lu, Rui Hu, Lin Peng, MengSi Liu, Zhen Sun

**Affiliations:** ^1^School of Pharmacy and Laboratory Science, Ya'an Polytechnic College, Ya'an, China; ^2^Hengyang Medical College, University of South China, Hengyang, China

**Keywords:** adalimumab, biosimilar, rheumatoid arthritis, tumor necrosis factor-α, efficacy, safety, immunogenicity

## Abstract

Adalimumab, as a TNF inhibitor biologic for the treatment of rheumatoid arthritis, is one of the top-selling drugs worldwide. As its various patents have gradually expired, experiments on its biosimilars are constantly being implemented. In this review, we summarized clinical trials of seven biosimilars currently approved by the FDA and/or EMA for the treatment of rheumatoid arthritis, namely: ABP 501 (Amjevita/Amgevita/Solymbic), BI 695501 (Cyltezo), SB5 (Imraldi/Hadlima), GP2017 (Hyrimoz/Hefiya/Halimatoz), MSB11022 (Idacio), FKB327 (Hulio), and PF-06410293 (Abrilada). Overall, these biosimilars showed similar efficacy, safety, and immunogenicity to adalimumab. All biosimilar switching trials indicated that switching from adalimumab to a biosimilar does not have a significant impact on efficacy, safety, and immunogenicity.

## Introduction

Rheumatoid arthritis (RA) is a chronic, inflammatory joint disease, which can lead to severe joint damage and disability, therefore decreasing the patient's quality of life. With the promotion of scientific research and with a more in-depth understanding of RA, more choices and better development in therapies have been achieved (1). Biologic disease-modifying anti-rheumatic drugs, for example, have been a major advance in the treatment of patients with RA (2).

As a cytokine that is central to the inflammatory cascade, tumor necrosis factor-alpha (TNF-α) regulates the immune response. Elevation of TNF-α levels have been observed in synovial fluid and the synovium of patients with RA (3, 4). It can lead to erosion of cartilage and bone destruction by inducing local inflammation and pannus formation and can then cause disability and loss of function. TNF-αinhibitors (TNFi), therefore, are indicated to treat moderately to severely active disease (5).

As the third TNFi approved by FDA, adalimumab (ADL) has shown excellent efficacy and safety and is widely used in clinical RA treatment (6). However, the long-term cost and high price is a primary defect (7). Therefore, the expected patent expiry of some of these therapeutics has stimulated interest in the development of biosimilars (8). A biosimilar is a biological medicine, that is similar, in terms of structure, function, and pharmacokinetics (PK), to another biological medicine that has previously been approved for use (9). Currently, seven ADL biosimilars are approved in the EU and/or the USA: ABP 501, BI 695501, SB5, GP2017, MSB11022, FKB327, and PF-06410293, all of which have been proven to be similar in terms of safety and efficacy to the licensed reference product (RP) (Table 1)^1^^,^^2^.

**Table 1:** Approval status of adalimumab biosimilars.

**Biosimilar name**	**Brand name**	**US FDA (approval status)**	**EMA (approval status)**
ABP 501	Amjevita/Amgevita/Solymbic	2016	2017
BI 695501	Cyltezo	2017	2017
SB5	Imraldi/Hadlima	2019	2017
GP2017	Hyrimoz/Hefya/Halimatoz	2018	2018
MSB11022	Idacio	-	2019
FKB 327	Hulio	2020	2018
PF-06410293	Abrilada/Amsparity	2019	2020

There are, however, still subtle differences between the immunogenicity, efficacy, and safety of the seven biosimilars. There is currently a lack of head-to-head experiments comparing the differences between these biosimilars. Thus, this review aims to summarize the efficacy, safety, and immunogenicity of each biosimilar by reviewing the data from clinical trials, to provide a reference for clinicians to choose the most suitable biosimilar for different RA patients.

## Results

### ABP 501 (Amjevita/Amgevita/Solymbic)

ABP 501 was the first ADL biosimilar to be approved by the FDA in 2016 and the EMA in 2017. In the preclinical study and phase I clinical trial, the function and PK was demonstrated to be similar between ABP 501 and ADL. In addition, the structural similarity that included general properties, primary and higher-order structure, carbohydrate structure, isoelectric profile, purity and impurities, and thermal-forced degradation profiles was also confirmed (10–12). The subsequent randomized, double-blind, phase III equivalence study was conducted in 526 patients with moderate to severe rheumatoid arthritis, despite methotrexate (MTX), to evaluate the safety, efficacy, and immunogenicity equivalence between ABP 501 and ADL (13).

The risk ratio of ACR20 at week 24 for the primary endpoint was 1.039 (90% CI: 0.954–1.133; ABP501:74.6%, ADL: 72.4%), which was within the predefined equivalence margin of 0.738 to 1.355

Furthermore, the mean change of DAS28-CRP comparing the baseline in both the ABP501 and ADL groups was −2.32, and the risk ratio for ACR50 and ACR70 was 0.948 (90% CI: 0.819–1.097; ABP 501: 49.2%, adalimumab: 52.0%) and 1.130 (90% CI: 0.872–1.464; ABP 501: 26.0%, adalimumab: 22.9%), further demonstrating the efficacy equivalence between ABP 501 and ADL.

The subsequent open-label extension (OLE) study evaluated the long-term safety and efficacy of APB 501 for rheumatoid arthritis patients, which included 467 patients who completed the parent study. At week 24, patients in the ABP 501 group continued with ABP501 (ABP501/ABP501) and patients in the ADL group transitioned to ABP 501(ADL/ABP 501). The ACR20 rates at week 48 and 70 were 77.6 and 78.8%, respectively. The ACR20 rates in the ABP 501/ABP 501 group and ADL/ABP 501 group were still comparable (14).

The treatment-emergent adverse events (TEAEs) rate of ABP 501 and ADL in the parent study was similar (50.0 and 54.6%, respectively), in which the percentage of patients with severe adverse events (SAEs) was also similar (3.8 vs. 5.0%, respectively). One malignant tumor was found in each group and no active tuberculosis was reported (13). In the OLE study, all the proportions of TEAEs (62.4 vs. 65.0%, respectively) and SAEs (10.9 vs. 8.9%, respectively) that occurred in the ABP 501/ABP 501 and ADL/ABP 501 group were similar (14). The most common adverse event in the short and long-term was infection. No fatal events were reported.

Antidrug antibody (ADA) status and neutralizing antibodies (nAbs) were assessed by a highly sensitive and drug tolerant assay based on the Meso Scale Discovery (MSD) electrochemiluminescence (ECL) platform and a cell-based bioassay using a TNFα-responding cell line that results in a TNFα-induced phosphorylation of nuclear factor κB, respectively (12). The percentage of patients with ABP 501 and the ADL groups that tested positive for binding ADAs (38.3 vs. 38.2%, respectively) and nAbs (9.1 vs. 11.1%, respectively) were similar. Of note, the percentage of ACR20 responders throughout the parent study was similar between the treatment groups despite ADA status (13). In the subsequent OLE study, the ADAs and nAbs were relatively increased, and the positive rate between those continuing on the ABP 501 group and those transitioning from ADL to the ABP 501 group remained similar (14).

### BI 695501 (Cyltezo)

BI 695501 was approved by the EMA and FDA in 2017. Previous research has confirmed its similarity in structure, function, and PK to ADL (15, 16).

A randomized, double-blind, parallel-arm, 58-week trial was conducted by Cohen et al. to demonstrate the clinical equivalence of BI 695501 with ADL. Six-hundred-and-forty-five patients with moderate-to-severe RA in this trial were all on stable MTX (15–25 mg/week background treatment for ≥12 weeks before enrolment and 10–14 mg/week was also permitted if patients were intolerant to larger dose). Patients were randomized 1:1 to receive 40 mg of BI 695501 (*n* = 324) or ADL (*n* = 321) subcutaneously, once every 2 weeks for 24 weeks. At week 24, patients were re-randomized to continue with the initial drugs or to switch from ADL to BI 695501(BI695501/BI695501, ADL/ADL and ADL/BI695501) (16).

Co-primary efficacy endpoints were ACR20 response at week 12 (requested by the FDA) and at week 24 (requested by the EMA), based on FAS. At week 12, 67.0 and 61.1% of patients in the BI 695501 group and ADL group achieved ACR20, respectively. The difference (5.9%; 90%CI −0.9 to 12.7) was within pre-specified margins set by the FDA to demonstrate equivalence: −12 and 15%. At week 24, 69.0 and 64.5% patients in the BI 695501 group and ADL group achieved ACR20, respectively. This difference (4.5%, 95%CI −3.4 to 12.5) was within −15 and 15% set by the EMA. The difference between BI 695501 and ADL was 4.3% (90%CI −2.8 to 11.3) at week 12 and 1.6% (90%CI– 5.3 to 8.5) at week 24, if based on the PPS. Secondary endpoints (ACR50, ACR70, EULAR responses, DAS28 and SF-36) were also reported to be similar at week 24. ACR20/50/70, DAS28-ESR, and EULAR were comparable at week 48, and no significant differences were reported, as well (16).

The safety analysis set (SAF) included all patients who received at least one dose of the trial drug. Again, overall safety was similar between the treatment groups. Up to week 58, patients with at least one TEAE were 193 (59.6%) in BI695501/BI695501, 93 (63.7%) in ADL/BI695501, and 105 (60.0%) in ADL/ADL. Among them, patients with at least one drug-related TEAE were BI695501/BI695501: 62 (19.1%), ADL/BI695501: 28 (19.2%) and ADL/ADL: 40 (22.9%). Among all TEAEs, infections and infestations were the most common organ class system: 35.2% (114/324) for continuous BI 695501 group vs. 34.3% (60/175) for continuous ADL. Up to week 24, serious infections all occurred in the ADL group: four had pneumonia, two had acute pyelonephritis, one had infective arthritis, one had appendicitis, and one had bronchitis. Cellulitis was reported in one patient in the BI 695501 group. From week 24 to week 58, in terms of serious infections, there was one patient with pneumonia in the ADL/ADL group, and one patient with influenza, viral pneumonia, and salmonella sepsis in the ADL/BI 695501 group. No deaths were reported (16).

ADA status was detected by a single bridging ECL assay based on the MSD platform with an acid dissociation step and nAbs was measured by a cell-based antibody-dependent cell-mediated cytotoxicity method. The comparable occurrence rates of patients developing ADA were 47.4% in the BI 695501 group and 53.0% in the ADL group, up to week 24. And this trial showed that nAbs frequencies between the BI 695501 and ADL group were also similar at week 24. Whether ADA or nAbs, they occurred in all three treatment groups at similar rates up to week 48, since the re-randomizing at week 24 (16).

### SB5 (Imraldi/Hadlima)

SB5 was approved by the EMA and FDA in 2017 and 2019, respectively, and was proven to have s similar structure, function, and PK to ADL (17, 18). Weinblatt et al. (17, 19) phase III, randomized, double-blind, parallel group study comparing SB5 with ADL in efficacy, safety, and immunogenicity included 544 moderate to severe RA patients, despite MTX treatment. In this trial, all patients were biologic naive previously. Patients were randomized 1:1 to receive 40 mg SB5 or ADL subcutaneously every other week for 24 weeks. At week 24, patients receiving ADL were randomized to SB5 or ADL until week 52, and patients on SB5 continued with SB5 (17, 19).

The primary efficacy endpoint was ACR20 response at week 24 in the per-protocol set (PPS; completer analysis). Results showed that the SB5 group and ADL group achieved comparable ACR20 response rates (72.4 and 72.2%, respectively), and the rate difference was 0.1% (95% CI, −7.83 to 8.13%). A similar ACR20 response was also observed in the full analysis set (FAS): 68.0% for the SB5 group and 67.4% for the ADL group. The rate difference was 0.8% (95% CI, −7.03 to 8.56%) for the FAS. The difference in both PPS and FAS were within the predefined equivalence margin (−15 to 15%). The ACR50 and ACR70 response rates at week 24 were equivalent and the mean change from baseline to week 24 in the DAS28-ESR scores was also comparable for SB5 and ADL (−2.74, −2.68, respectively). Other secondary efficacy endpoints were all comparable according to the results (17). A subsequent transition study showed an equivalent ACR response at week 52 in the SB5/SB5, ADL/SB5, and ADL/ADL group, indicating that a transition from ADL to SB5 did not affect the efficacy and the long-term efficacy of SB5 (19).

TEAEs occurred in 35.8% of patients in the SB5 group and in 40.7% of patients in the ADL group by week 24. Among them, 10.1 and 11.7% of patients were considered related to the study drug, respectively. 1.1% patients in the SB5 group and 2.9% patients in the ADL group were reported to have serious TEAEs. One (0.4%) patient in the SB5 group (Escherichia urinary tract infection) and two (0.7%) patients in the ADL group (bronchopneumonia and staphylococcal sepsis) presented serious infections. The proportion of patients reported to have injection site reactions was similar between the SB5 and ADL group. Two patients in the ADL group experienced malignancy (lymphoma and metastases to spine, papillary thyroid cancer). Two deaths occurred up to week 24 and were not considered to be related to the study drug (17). The safety was also comparable between the SB5/SB5, ADL/SB5, and ADL/ADL groups after switching at week 24: the proportion of any TEAMs was 32.3, 37.6, and 33.1%, respectively (19).

ADA status was determined using MSD ECL bridging, applying an SB5 single tagged immunoassay. At week 24, the incidence of ADA was similar for the SB5 and ADL groups (33.1 and 32.0%, respectively). 32.4% (80/247) and 31.4% (82/261) of patients were reported to have emergent ADAs in the SB5 group and ADL group. And the proportion of boosted Abs was 42.1% (8/19) and 50% (4/8), respectively. In both treatment groups, about half of all the antibodies were found to be neutralizing. The incidence at week 52 was also comparable after switching: 15.7% (40/254), 16.8% (21/125), and 18.3% (23/126) in the SB5/SB5, ADL/SB5, and ADL/ADL groups, respectively (17, 19).

### GP2017 (Hyrimoz/Hefya/Halimatoz)

GP2017 was approved by the FDA and EMA in 2018. Previous studies demonstrated that GP2017 and Humira have identical amino acid sequences, indistinguishable secondary and tertiary structures, the same level of post-translational modifications, and a functional and pharmacological similarity to the reference drug (20, 21). Wiland et al. (22, 23) conducted a phase III trial including 353 moderate-to-severe RA patients with inadequate response to disease modifying anti-rheumatic drugs. Patients were on a stable dose of MTX with biologics. At week 24, the patients in the ADL group were switched to receive GP 2017 up to week 46 (22, 23).

The primary endpoint mean change of DAS28-CRP from baseline to week 12 was −2.16 in the GP2017 group and −2.18 in the ADL group (RD = 0.02; 95% CI: −0.24, 0.27). The mean change of DAS28-CRP from baseline to week 48 in the FAS population was −2.92 and −2.74 in the ADL /GP2017, and GP2017/GP2017 groups, respectively. In addition, the ACR20/50/70 responses of the two groups were also similar before and after switching, throughout the trial (22, 23).

Respectively, 61.6 and 60.2% of patients in the GP2017 and ADL groups presented with TEAEs and the most common TEAE was infections and infestations. During the switch period, the incidence of TEAEs were 32.5 and 36.5% in the ADL /GP2017 and GP2017/GP2017 groups, respectively. The incidence of SAEs was low and remained comparable in both groups during the entire study (ADL /GP2017: 5.7%; GP2017/GP2017: 4.0%), and no deaths occurred (22, 23).

A validated competitive ligand-binding assay and an ECL bridging immunogenicity assay were used to assess the incidence of ADAs. ADAs tested positive in 24.2 and 25.6% of patients in the GP2017/continued and ADL/switched group, respectively, of which more than 70% in both groups had a positive nAbs. After switching, 26.3 and 24.0% of patients tested positive for ADAs in the ADL/switched and GP2017/continued groups, respectively. No statistical differences were observed. The ADA status did not have a clinically significant impact on safety (22, 23).

### MSB11022 (Idacio)

MSB11022 was approved by the EMA in 2019. Preclinical studies have proven its structural and functional similarities to the reference drug, which included the same amino acid sequence, N-/C-terminal modifications, the relative distribution of the intact and glycated forms of both light chain and heavy chain, as well as C-terminal lysine truncation of a heavy chain level of MBS11022. Lower oxidation levels showed in MSB11022 compared to ADL. There was also no difference in the high-order structure (24). A phase I trial conducted in healthy subjects has demonstrated bioequivalence with the reference drug in terms of PK, safety, tolerability, and immunogenicity (25). Edwards et al. (26) conducted a phase III randomized, double blind parallel group, 52-week trial which included 288 patients with moderately-to-severely active RA on stable MTX treatment with an inadequate response. The phase III trial consisted of a 4-week screening period, a 52-week double-blind treatment period, and a 4-month safety follow-up period (26).

The ACR20 response rates at week 12 for the ITT population were 79.6 and 80.9% for MSB11022 and ADL, respectively. The 95% CI for treatment difference was −10.55 to 8.04, which demonstrated the similarity between the biosimilar and reference product. The similarity persisted up to week 52. The proportion of patients achieving ACR50 and ACR70, DAS28- ESR scores, SDAI, and CDAI scores were also similar between the two treatments throughout the trial (26).

By week 52, the percentage of TEAEs was similar between the MSB11022 group and ADL group (58.0 vs. 64.1%, respectively), while compared with the ADL group, patients in the MSB11022 group had a lower rate of permanent discontinuation due to TEAEs (4.2 vs. 9.7%). In addition, compared with the reference product, MSB11022 also showed a trend toward reduced incidence of serious TEAEs (9.7 vs. 4.9%, respectively) and treatment-related TEAEs (40 vs. 21.7%, respectively). Hypersensitivity, the primary endpoint of the trial occurred in similar proportions and types in both the MSB1022 and ADL groups (4.2 vs. 5.5%, respectively). There were fewer injection site reactions (ISRs) in the MSB11022 group than in the ADL group (9.1 vs. 22.8%, respectively). These differences were not considered notable (26).

An ECL bioanalytical method based on the MSD platform was used to detect the ADA status. Respectively, 80.4 and 71.7% of patients in the MSB11022 and ADL groups had at least one positive ADA result, of which 39.9% in the MSB11022 group and 39.3% in ADL group had a positive nAb result. Of note, The ADA-positive patients were observed to have a lower mean trough concentration than the ADA-negative patients, but no differences in efficacy and safety were reported (26).

Although switching treatment between MSB11022 and RP is not currently performed in RA patients, the switching effects have been evaluated in patients with psoriasis. In the phase III AURIEL-PsO trial, Psoriasis patients with a more than 50% improvement in the Psoriasis Area and Severity Index in the RP treatment group were rerandomized 1:1 to continue RF or to switch to MSB11022. Their results showed that the time to reach PASI 75, 90, and 100 and Physician's Global Assessment improvements in the three treatment groups at week 24 and 52 were comparable. The safety endpoints were also similar across the three groups, including serious adverse events, treatment-related TEAEs, and treatment discontinuations due to TEAEs. The incidences of a positive ADA result in the RP/RP, MSB11022/MSB11022, and RP/MSB11022 group were 92.1, 93.2, and 94.1%, respectively, and about 60% of patients were nAD-positive, which did not show statistical difference. In addition, the PK results were comparable after switching. In conclusion, the trial found that switching did not affect the endpoints of interest in psoriasis patients, which provides supporting evidence for its use in RA patients (27).

Currently, MSB11022 has two formulations: one is a citrate buffer, the other is an acetate buffer. The phase III trial only studied the acetate-buffered formulation, but an additional study demonstrated bioequivalence and a similar safety and immunogenicity profile between two formulations in healthy subjects (EMR200588-003).

### FKB327 (Hulio)

FKB327 was approved by the EMA and FDA in 2018 and 2020, respectively. A preclinical trial has demonstrated its structural and functional similarity to ADL. A subsequent phase I trial has demonstrated similarities in PK and safety in healthy subjects (28)^3^. Genovese et al. conducted a randomized, phase III, double-blind study, and its open-label extension, which enrolled 730 active rheumatoid arthritis patients aged ≥18 years on a stable dose of MTX. In this trail, patients were randomized 1:1 to receive FKB327 or RP for 24 weeks in period 1, and then the completed patients were randomized 2:1 according to the original group, two-thirds of which continued the same treatment, and one-third of which switched until week 54 in period 2, and then a double switch was performed in the FKB327 to the ADL group and the continuing ADL group was switched to FKB327 in period 3 (29, 30).

The primary efficacy endpoint was the treatment difference of ACR20 at week 24 between the FKB327 and ADL groups, and its 95% CI was −7.9 to 4.7 (FKB327: 74.1%, ADL: 75.7%) and the 95% CI of the least squares mean DAS28-CRP difference at the same time was −0.16 to 0.18 (FKB327: 3.43, RP: 3.42), all of which were within the predefined equivalence margin and all of which demonstrated their efficacy similarity. In addition, the percentages of patients achieving ACR50 and ACR70 were comparable for the two treatments throughout period 1, and the subgroup analyses were performed by geographic region, DAS28-CRP concentration, and prior biologic medication for RA, which did not show differences among these subgroups. The subsequent OLE trial also indicated similar increases in ACR20, ACR50, or ACR70 response rates and decreases in mean DAS28-CRP in four treatment groups at all visits, which demonstrated that the efficacy was not affected by switching treatments. The efficacy tended to increase over time throughout the trial (29, 30).

The treatment-emergent adverse events (TEAEs) were similar, with more than 60% in period 1 and period 2, and the long-term TEAEs was also comparable in patients treated with FKB327 and patients treated with the RP (1.707 vs. 2.075 events per patient-year, respectively). The treatment-emergent serious AEs were also similar but the percentage of treatment-emergent SAEs resulting in discontinuation were relatively higher for FKB327 than for RP (0.025 vs. 0.011 events per patient-year, respectively). The most common TEAEs were nasopharyngitis and other infections. Of note, latent TB was reported to be one of the most common adverse events, but it was thought to be the result of the included population, which included patients from counties with general high TB rates. The ISR rates in all of groups were low and comparable. The incidences of malignancy or death were low, and each group was balanced without meaningful differences (29, 30).

ADA status was determined using the ECL bridging format based on MSD and an acid dissociation was introduced. A sensitive competitive ligand binding method was used to detect nAbs. The percentage of ADA-positive patients at week 24 was 57.9% in the FKB327 group and 55.5% in the RP group, in which almost all patients were neutralizing ADAs. At week 54, the percentage of RP/RP, FKB327/FKB327, RP/FKB327, and FKB327/RP groups were 51.6, 52.2, 45.2, and 61.0% respectively, with most of them remaining with neutralizing ADAs. The slight differences across all treatment groups were deemed to be associated with the smaller number of patients in each group after re-randomization. The percentage of patients who tested positive for ADAs and titer level in all treatment groups were comparable (29).

### PF-06410293 (Abrilada, Amsparity)

PF-06410293 was approved by the FDA and EMA in 2019 and 2020, respectively. Peptide mapping analysis showed that PF-06410293 and ADL had identical amino acid sequences (31). Efficacy, safety, tolerability, and immunogenicity profiles of PF-06410293 have also been proven to be similar to ADL in previous studies (31, 32).

A double-blind, randomized, study evaluating the efficacy, safety, immunogenicity, PK, and pharmacodynamics of PF-06410293597 vs. ADL was conducted by Fleischmann et al. Eligible RA patients were randomized 1:1 to receive 40 mg PF-06410293 (*n* = 297) or ADL (*n* = 300) once every 2 weeks. Patients were on a stable dose of MTX with biologics. At week 26, treatment period 2 was started, patients receiving ADL were re-randomized 1:1 to receive PF-06410293 or to continue with ADL for 26 weeks, while patients in the PF-06410293 group remained on the PF-06410293 treatment (33).

The primary endpoint was ACR20 response at week 12. At week 12, 68.7% (204/297) of patients in the PF-06410293 group and 72.7% (218/300) patients in the ADL group achieved ACR20, and the difference was −3.98%. With non-responder imputation, the treatment difference at week 12 in ACR20 (−2.98%; 95%CI −10.38 to 4.44 and 90%CI −9.25 to 3.28%) was within the pre-specified margins (95%CI ± 14% and 90%CI −12 to 15%) demonstrating equivalence between PF-06410293 and ADL. This study also showed similar ACR20 results between PF-06410293 and ADL in the PPS (71.1 vs. 75.2%). Secondary endpoints were also comparable between treatment groups at week 12 (33). In treatment period 2, ACR20 and other endpoints remained similar between the treatment groups (34).

In treatment period 1, the proportions of patients with at least one TEAE were similar: 48.1% (143/297) in the PF-06410293 group and 47.8% (143/299) in the ADL group. The most frequently reported TEAEs were increased alanine aminotransferase, viral upper respiratory tract infections, hypertension, and headaches. No active TB occurred. SAEs were 4.0 and 4.3% in the PF-06410293 and ADL group, respectively. The system organ classes with the highest proportion of patients with SAEs were infestations and infections, which occurred in three patients in each treatment group. The incidence of injection-site reactions (1.7 and 2.0%) and opportunistic infections (2.4 and 1.7%) was similar between treatment groups (33). In treatment period 2, after switching at week 26, adverse events occurred at 43.5% (123/243) in the PF-06410293/PF-06410293 group, 44.4% (60/112) in ADL/ADL group, and 38.3% (51/100) in ADL/ PF-06410293 group. Other results were also similar (34).

A single validated ECL immunoassay was used to determine the ADA status, and then the ADA-positive patients were further tested for nAbs activity with a validated cell-based assay using PF-06410293 as the capture agent. Overall, 44.4 and 50.5% of patients in the PF-06410293 and ADL group tested positive for ADA at least once, respectively, and 13.8 and 14.0% of patients developed nAbs in the PF-06410293 and ADL group, respectively (33). Incidences of ADA through week 52 were also comparable between treatment groups with 47.3, 54.1, and 45.9% for the PF-06410293/PF-06410293, ADL/ADL, and ADL/ PF-06410293 group, respectively (34).

## Discussion

**Figure 1:**
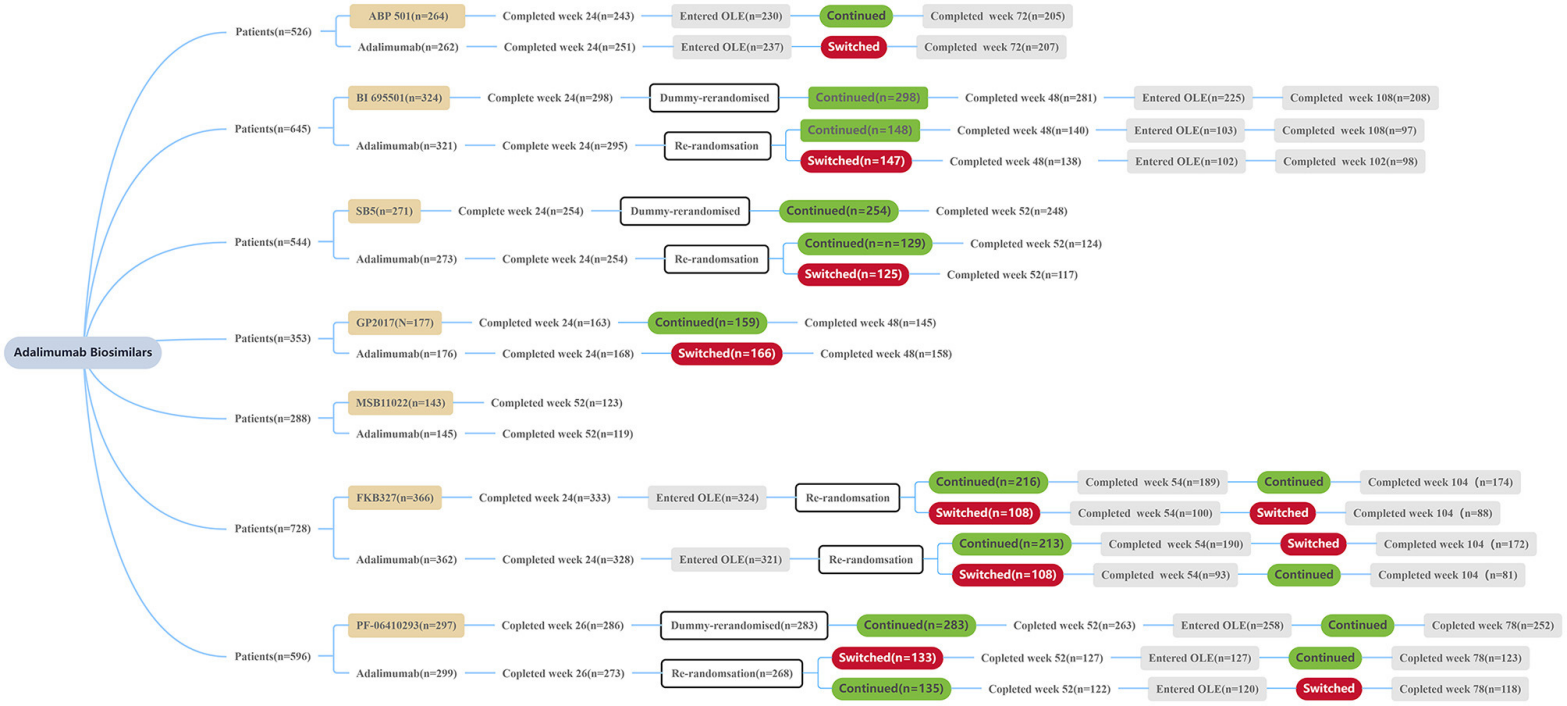
Patient disposition summary of each trial. All patients received ADL or a biosimilar at a dose of 40 mg/0.8 mL solution subcutaneously every other week during the treatment period. OLE, open label extension.

Each clinical trial showed that currently approved ADL biosimilars and RP had similar endpoints, including in the switching treatment performed (Table 2) (Figure 1). In clinical practice, to switch from ADL to a biosimilar, it must be based on a shared decision between the patients and the prescribing physician, and post-marketing safety analyses. It is worth noting that if a biosimilar gets the “interchangeability” designation allowed by the FDA, the biosimilar could be automatically substituted at the pharmacy level without consulting the prescribing physician (35). This designation can be applied only if the manufacturer is able to provide sufficient evidence that “the risk in terms of safety or diminished efficacy of alternating or switching between use of the biological product (biosimilar) and the RP is not greater than the risk of using the RP without such alternation or switch.” Yet, none of the biosimilars have received this designation (36). These biosimilars may become substitutes of brand biological agents. Furthermore, along with the similarity to ADL and its lower price, biosimilars have the opportunity to make biologic treatment for RA more widely available. At the moment, however, this goal has not been achieved due to a number of reasons such as high prices, for example (37, 38). An important limitation of this review is the relative lack of real-life studies of the contrast between ADL and its biosimilars. More extensive and in-depth real-life research is required to allow for biosimilars to become available.

**Table 2:** The summary of characteristic and key endpoints of each pivotal trial.

**Biosimilar name**	**Patients**	**Time of treatment^†^**	**Incidence of TEAEs vs. ADL**	**RR (95%CI)**	**Primary efficacy endpoint vs. ADL**	**RR (95%CI)**	**ACR70 response vs. ADL**	**RR (95%CI)**	**Incidence of ADAs vs. ADL**	**RR (95%CI)**
ABP 501 (NCT01970475) (NCT02114931)	18–80 years old with moderate to severe active RA despite MTX	68 weeks	50.0 vs. 54.6% up to week 26	0.92 (0.78–1.08)	ACR20: 74.6 vs. 72.4% at week 24	1.03 (0.93–1.14)	26.0 vs. 22.9% at week 24	1.14 (0.83–1.55)	38.3 vs. 38.2% up to week 26	1.00 (0.81–1.25)
BI 695501 (NCT02137226) (NCT02640612)	18–80 years old with moderate to severe active RA despite MTX	98 weeks	59.6 vs. 60.0% up to week 58	0.99 (0.85–1.15)	ACR20: 67.0 vs. 61.1% at week 12, 69.0% vs. 64.5% at week 24	1.10 (0.98–1.23), 1.07 (0.96–1.19)	10.0 vs. 11.0% at week 12, 13.4 vs. 18.2% at week 24	0.91 (0.58–1.43), 0.73 (0.51–1.06)	47.7 vs. 53.0% up to week 24	0.90 (0.76–1.05)
SB5 (NCT02167139)	18–75 years old with moderate to severe active RA despite MTX	52 weeks	35.8 vs. 40.7% up to week 24	0.88 (0.71–1.09)	ACR20: 72.4 vs. 72.2% at week 24	1.00 (0.90–1.12)	19.2 vs. 20.3% at week 24	0.95 (0.66–1.37)	33.1 vs. 32.0% up to week 24	1.03 (0.80–1.33)
GP2017 (NCT02744755)	≥18 years old with moderate to severe active RA despite MTX	46 weeks	61.6 vs. 60.2% up to week 24	1.02 (0.87–1.21)	DAS28-CRP: −2.16 vs. −2.18 at week 12 ACR20: 82.4 vs. 78.6% at week 12	0.02^*^ (−0.24 to 0.27) 1.05 (1.92–1.19)	21.3 vs. 26.2% at week 12	0.81 (0.51–1.30)	NP	NA
MSB11022 (NCT03052322)	≥18 years of age with moderate to severe active RA despite MTX	48 weeks	58.0 vs. 64.1% up to week 52	0.90 (0.75–1.10)	ACR20: 79.6 vs. 80.9% at week 12	0.98 (0.88–1.11)	27.3 vs. 20.0% at week 12	1.36 (0.89–2.08)	80.4 vs. 71.7% up to week 52	NA
FKB327 (NCT02260791) (NCT02405780)	≥18 years of age with moderate to severe active RA despite MTX	100 weeks	62.2 vs. 66.2% up to week 54	0.94 (0.85–1.03)	ACR20: 74.1 vs. 75.7% at week 24	0.98 (0.90–1.07)	21.3 vs. 25.1% at week 24	0.84 (0.65–1.10)	57.9 vs. 55.5% up to week 24	1.04 (0.92–1.18)
PF-06410293 (NCT02480153)	≥18 years of age with active RA despite MTX	78 weeks	48.1 vs. 47.8% up to week 26	1.01 (0.85–1.19)	ACR20: 68.7 vs. 72.7% at week 12	0.95 (0.85–1.05)	16.5 vs. 19.0% at week 12	0.87 (0.61–1.23)	44.4 vs. 50.5% up to week 26	0.88 (0.74-1.04)

† *Time of treatment included the last time the patients in the open label extension trial received the test drug*.

* *mean difference*.

*RR, risk ratio; TEAEs, treatment-emergent adverse events; ADL, adalimumab; ADAs, anti-drug antibodies; NP, not provided; NA, not available (because of the lack of data cannot be calculated)*.

The immunogenicity of biologics that can lead to the occurrence of ADAs and nAbs in patients, and therefore affect PK, alter clinical safety and efficacy profiles, and is an important consideration of biosimilar studies (39). We reviewed several possible factors that may confound the results of immunogenicity and noticed some limitations in the assessment of immunogenicity. First, concomitant medication may affect the incidence of ADAs and nAbs. In the phase III trials of all seven biosimilar studies presented in this review, patients used MTX while being treated with biologics, but the combination of MTX and biologics can lead to therapeutic protein-drug interactions, which can then reduce the incidence of ADAs and improve efficacy (40, 41). Second, most of the trials allowed patients to be treated with <2 biologic therapies (such as etanercept, infliximab) other than ADL and its biosimilars prior to the start of the trial, which may have a potential impact on the incidence of ADAs and nAbs. Third, the incidence of ADAs and nAbs increased with the duration of treatment. Furthermore, an ECL assay that has high sensitivity, and a broad dynamic range was used to detect the ADAs status in all trials, however, most of them did not consider the steps (such as acid dissociation pre-treatment) to disrupt circulating ADAs/nAbs-drug complexes, which may underestimate the incidence of ADA-positivity in each arm and ADA titer. In addition, it may be affected by the matrix effect in the test sample. In terms of the detection of nAbs, cell-based assays, which can test multiple functional domains and better mimic the mechanisms by which nAbs work their effect in living biological systems, are generally the preferred method (42). But the cell-based assays were only used in the trials of ABP501, BI 695501, and PF-06412393.

## Conclusion

Based on the pivotal trials of the seven biosimilars, all of them showed comparable efficacy, safety, and immunogenicity to ADL. Subtle differences are considered to be due to some methodological bias, rather than the properties of biosimilars, however, several limitations in immunogenicity assessment are of concern. For the biologic-naïve patients, biosimilars may be chosen for the first biologic therapy of RA. Second, all biosimilar studies conducted research on the transition from ADL to biosimilars. The results of these studies have shown that switching from ADL to a biosimilar does not have a significant impact on efficacy, safety, and immunogenicity.

## Author Contributions

ZS and RH contributed to the conception and design of the study. RH, LP, ML, and XL searched the database and extract data. ZS, ML, and LP made tables. ML and XL made figures. RH, ZS, and XL wrote the first draft of the manuscript. All authors contributed to manuscript revision, read, and approved the submitted version.

## Conflict of Interest

The authors declare that the research was conducted in the absence of any commercial or financial relationships that could be construed as a potential conflict of interest.

## Footnotes

^1^Generics and Biosimilars Initiative (GaBI). *Biosimilars Approved in Europe*. Available online at: http://www.gabionline.net/Biosimilars/General/Biosimilars-approved-in-Europe (accessed December 18, 2020).

^2^Generics and Biosimilars Initiative (GaBI). *Biosimilars Approved in the US*. Available online at: http://www.gabionline.net/Biosimilars/General/Biosimilars-approved-in-the-US (accessed December 18, 2020).

^3^European Medicines Agency. *Human Medicine European Public Assessment Report: Hulio*. Available online at: https://www.ema.europa.eu/en/documents/assessment-report/hulio-epar-public-assessment-report_en.pdf (accessed November 25, 2020).

## Abbreviations

ADA: antidrug antibody
ADL: adalimumab
CPR: C reactive protein
ESR: erythrocyte sedimentation rate
ECL: electrochemiluminescence
FAS: full analysis set
ISR: injection site reaction
MSD: Meso scale discovery
MTX: methotrexate
OLE: open-label extension
PPS: per-protocol set
PK: pharmacokinetic
RP: reference product
SAE: severe adverse event
SAF: safety analysis set
TEAE: treatment-emergent adverse event
ACR: American College of Rheumatology
20: 50 or 70% improvement in the ACR core set measurements
DAS28: 28-joint disease Activity Score
DAS28: is considered positive when remission <2.6
EULAR: European league against rheumatism criteria (based on changes in DAS28).
